# Sexual Dysfunction and the Impact of Beta-Blockers in Young Males With Coronary Artery Disease

**DOI:** 10.3389/fcvm.2021.708200

**Published:** 2021-07-21

**Authors:** Yuxiang Dai, Zhendong Mei, Shuning Zhang, Shalaimaiti Shali, Daoyuan Ren, Lili Xu, Wei Gao, Shufu Chang, Yan Zheng, Juying Qian, Kang Yao, Junbo Ge

**Affiliations:** ^1^Department of Cardiology, Zhongshan Hospital, Shanghai Institute of Cardiovascular Disease, National Clinical Research Center for Interventional Medicine, Shanghai, China; ^2^State Key Laboratory of Genetic Engineering, School of Life Sciences, Human Phenome Institute, Fudan University, Shanghai, China; ^3^Ministry of Education Key Laboratory of Public Health Safety, School of Public Health, Fudan University, Shanghai, China

**Keywords:** early onset of coronary artery disease, erectile dysfunction, revascularization, beta-blockers, Chinese males

## Abstract

**Objective:** We aimed to assess the association of erectile dysfunction (ED) with the extent of coronary atherosclerosis, and to examine whether revascularization and medication use have an impact on ED status in patients with early onset of coronary artery disease (EOCAD).

**Methods:** International Index of Erectile Function (IIEF-5) was used to evaluate sexual function in 296 male patients with EOCAD (age, 39.9 ± 4.8 years), and 354 male controls (age, 40.6 ± 4.4 years). The extent of coronary atherosclerosis was measured by Gensini score. Endothelial function was evaluated by two vasomotor indexes including endothelin-1 (ET-1) and nitric oxide (NO) by ELISA.

**Results:** ED was more frequent (57.8 vs. 31.1%, *P* < 0.001) and serious (IIEF-5 score:17.7 ± 6.0 vs. 21.6 ± 5.0, *P* < 0.001) among EOCAD patients than that among controls. IIEF-5 score was negatively correlated with Gensini score (r = −0.383, *P* < 0.001). The adjusted odds ratio (OR) for the presence of ED (EOCAD vs. controls) was 1.88 [95% confidential interval (CI), 1.12-3.18]. However, ET-1 and NO attenuated the association between ED and EOCAD (adjusted OR: 1.54, 95% CI: 0.84-2.80). IIEF-5 score increased after coronary revascularization in patients not on beta-blockers (18.71 ± 4.84 vs. 17.59 ± 6.05, *P* < 0.001) as compared with baseline, while stayed unchanged in the subgroup using beta-blockers (17.82 ± 5.12 vs. 17.70 ± 5.98, *P* = 0.09).

**Conclusions:** ED was common in patients with EOCAD, and associated with the severity of coronary atherosclerosis. Endothelial dysfunction may be a pathophysiologic mechanism underlying both ED and EOCAD. Coronary revascularization confers a benefit in ED amelioration, while this effect did not appear in patients using beta-blocker.

## Introduction

Coronary artery disease (CAD) is the most commonly diagnosed heart disease and the leading cause of death in adults, predominantly the elderly (1). The growing number of cases of early onset of coronary artery disease (EOCAD) over the past decades has raised major concern, especially in developing countries such as China, where CAD-related morbidity and mortality continue to rise despite notable advances in diagnostic and treatment capabilities (2). Depression and unhealthy lifestyles possibly play crucial roles in EOCAD, which is associated with increased risk of heart failure, cardiac death, impaired quality of life, and reduced work capacity with their socioeconomic consequences (3). Although adults aged ≤ 45years tend to be more sexually active, erectile dysfunction (ED), defined as persistent inability to achieve and maintain an erection sufficient to provide adequate sexual activity (4), is now increasingly common and an important cause of poor quality of life and psychosocial morbidity in this population (5, 6). Nonetheless, little is known about the prevalence of ED in patients with EOCAD, and coronary atherosclerotic burden has not been previously linked to ED severity. The present study therefore tested the hypotheses that the prevalence of ED is higher in patients with EOCAD as compared with the age-matched control group and the severity of ED is associated with the extent of coronary atherosclerosis. We further examined whether revascularization and beta-blockers have an impact on ED symptoms in EOCAD patients.

## Methods

Study subjects consisted of individuals enrolled in the prospective, multicenter, observational GRAND (Genetics and clinical characteristics in coRonary Artery disease in ChiNese young aDults) study. A total of 296 male cases with CAD aged 18-45 years, and 354 age-matched male controls were enrolled in the present study. Patients with EOCAD had a minimum of one major epicardial coronary artery (left anterior descending artery, left circumflex artery, or right coronary artery) with ≥50% stenosis documented by coronary angiography (CAG), and underwent coronary revascularization. Controls did not meet the latter CAG criteria. Patients and controls unwilling to answer the International Index of Erectile Function (IIEF-5) questionnaire (7, 8) were excluded. The investigation conformed to the principles outlined in the Declaration of Helsinki. All subjects provided informed consent, and the study protocol was approved by the ethical committee of the Zhongshan Hospital.

Clinical data were derived from the electronic medical records (EMR) of hospitalized patients. Each patient completed a detailed questionnaire on lifestyle and medical history before CAG. Risk factors analyzed included age, sex, body mass index (BMI), obesity, smoking, drinking, hypertension, hyperlipidemia, atrial fibrillation, and diabetes mellitus. Laboratory data were collected upon admission to the hospital, including hemoglobin, neutrophil to lymphocyte ratio (NLR), red cell distribution width (RDW), platelet distribution width (PDW), alanine transaminase (ALT), serum creatinine (Crea), estimated glomerular filtration rate (eGFR) calculated by the abbreviated MDRD equation, uric acid (UA), C reative protein (CRP), glycosylated hemoglobin (HbA1c), total cholesterol (TC), triglycerides (TG), low-density lipoprotein cholesterol (LDL-C), high-density lipoprotein cholesterol (HDL-C), apolipoprotein A1 (Apo A1), apolipoprotein B (Apo B), apolipoprotein E (Apo E). Left ventricular end-systolic dimension (LVESD), left ventricular end-diastolic dimension (LVEDD), and left ventricular ejection fraction (LVEF), and other parameters, were assessed by transthoracic echocardiography using the Teichholz method prior to CAG.

Peripheral venous blood (2 ml) was collected from all enrolled subjects before angiography. The serum samples were stored at −80°C until analysis. Serum levels of endothelin-1 (ET-1) and nitric oxide (NO) was detected by the ELISA method using endothelin-1 quantikine ELISA kit (R&D Systems Inc., Minneapolis, USA) and total nitric oxide and nitrate/nitrite parameter assay kit (R&D Systems Inc., Minneapolis, USA) respectively.

Medications, including beta-blockers, aspirin, clopidogrel, statins, diuretics, angiotensin-converting enzyme inhibitors (ACEI), angiotensin receptor blocker (ARB), calcium channel blockers (CCB), nitrates, oral hypoglycemic, and insulin, were evaluated through reviewing EMRs and by personal interviews before CAG and after discharge.

CAG analysis was performed by the cardiologist who was unaware of the patient's condition of ED. The lesions were analyzed in multiple projections. Gensini score was calculated to assess the coronary atherosclerotic severity. Each coronary stenosis was assigned a score according to the degree of luminal narrowing (stenosis of 25, 50, 75, 90, 99%, and complete occlusion were assigned a Gensini score of 1, 2, 4, 8, 16, and 32, respectively). The score was then multiplied by the coefficient according to the functional importance of the myocardial area supplied by that segment (9).

Erectile function was evaluated by using IIEF-5, which is an internationally validated self-administered assessment designed to quantitatively evaluate the patient's erectile function through a five-item short-form questionnaire (7, 8). The sum score ranges from 5 to 25 points with lower values corresponding to decreased erectile function. The severity of ED was classified into five levels based on the scores: severe (5–7), moderate (8–11), mild to moderate (12–16), mild (17–21), and no ED (22–25) (7, 8). Each subject was followed by telephone interview and the IIEF-5 questionnaire was reassessed at the 1-year follow-up timepoint.

Data are presented as mean ± standard deviation (SD) for continuous variables and percentage (%) for categorical variables. Continuous variables were compared between cases and controls using *t*-test. Categorical variables were compared using the chi-square test. The Pearson correlation coefficient was used to indicate correlations between quantitative variables. The independent association between ED status and the occurrence of EOCAD was determined by using a multivariable logistic regression model. The odds ratio (OR) and 95% confidence interval (CI) were calculated. Covariates were identified if the *P*-value was < 0.1 in univariate analyses. Changes in IIEF-5 score after coronary revascularization were analyzed by linear mixed-effects models after adjustment for the identified covariates. Hypothesis testing was two-tailed and a *P*-value < 0.05 was considered statistically significant. All the statistical analyses were performed using R version 3.5.1 (https://www.r-project.org/).

## Results

### Baseline Characteristics

The baseline characteristics of the 296 patients with EOCAD and 354 control subjects are provided in Table 1. Patients and controls were well-matched for age (mean of ~40 years for both) and had similar distributions of history of hypertension, diabetes mellitus, atrial fibrillation, values of echocardiographic parameters (including LVEF, LVESD, and LVEDD), and hemoglobin level, RDW, PDW, ALT, Creatine, eGFR, and CRP. However, the proportions of individuals with smoking and (or) drinking habits or hyperlipidemia, and the levels of BMI, NLR, UA, HbA1c, TC, TG, LDL-C, ApoB, non-HDLC, and the ratio of TG/HDL-C were considerably higher in the EOCAD group, while the levels of HDL-C and ApoA1 were lower as compared with controls (Table 1).

**Table 1:** Baseline clinical characteristics of EOCAD patients and controls.

	**EOCAD group (*N* = 296)**	**Control (*N* = 354)**	***P*-value**
**Demographic data**			
Age (years)	39.9 (±4.8)	40.6 (±4.4)	0.065
BMI (kg/m^2^)	29.2 (±9.1)	26.3 (±4.8)	<0.001
Obesity			0.128
Normal	81 (27.4%)	108 (30.5%)	
Overweight	94 (31.8%)	115 (32.5%)	
Obese	116 (39.2%)	106 (29.9%)	
**Lifestyle**			
Smoking			<0.001
Never	80 (27.0%)	187 (52.8%)	
Ever	111 (37.5%)	91 (25.7%)	
Current	104 (35.1%)	76 (21.5%)	
Drinking			<0.001
No	117 (39.5%)	264 (74.6%)	
Mild	138 (46.6%)	38 (10.7%)	
Severe	31 (10.5%)	51 (14.4%)	
**Coronary angiographic evaluation**			
Gensini score	82.4 (±54.8)	1.7 (±2.6)	<0.001
**Medical history**			
Hypertension	114 (38.5%)	136 (38.4%)	0.563
Hyperlipidemia	49 (16.6%)	33 (9.3%)	0.003
Atrial fibrillation	7 (2.4%)	8 (2.3%)	1.000
Diabetes mellitus	37 (12.5%)	39 (11.0%)	0.449
**Echocardiography parameters**			
LVEF (%)	59.9 (±9.0)	61.3 (±10.1)	0.058
LVSED (mm)	33.0 (±6.4)	33.6 (±7.3)	0.252
LVDED (mm)	50.6 (±5.6)	50.6 (±6.9)	0.983
**Biochemical measurements**			
HgB (g/L)	146.0 (±15.1)	146.6 (±17.8)	0.688
Neutrophil to lymphocyte ratio (NLR)	2.6 (±2.2)	2.2 (±1.9)	0.024
RDW (%)	12.4 (±1.3)	12.5 (±2.2)	0.975
PDW (%)	13.4 (±3.2)	13.2 (±3.3)	0.505
ALT (U/L)	38.3 (±25.4)	39.8 (±63.5)	0.691
Crea (μmol/L)	85.6 (±19.1)	84.4 (±25.0)	0.483
eGFR (ml/min/1.73m^2^)	100.1 (±17.1)	99.8 (±16.2)	0.849
UA (μmol/L)	427.6 (±93.1)	387.0 (±87.2)	<0.001
CRP (mg/L)	10.5 (±31.0)	7.4 (±24.5)	0.199
HbA1c (%)	6.1 (±1.4)	5.8 (±1.0)	0.018
TC (mmol/L)	4.45 (±1.46)	4.22 (±1.00)	0.028
TG (mmol/L)	2.70 (±1.64)	2.31 (±1.97)	0.007
HDL-C (mmol/L)	0.92 (±0.28)	1.01 (±0.26)	<0.001
LDL-C (mmol/L)	2.55 (±1.34)	2.3 (±0.9)	0.019
Apo A1 (g/L)	1.15 (±0.20)	1.22 (±0.23)	<0.001
Apo B (g/L)	0.92 (±0.31)	0.87 (±0.25)	0.034
Apo E (mg/L)	46.2 (±25.4)	41.9 (±17.4)	0.016
NHDL-C (mmol/L)	3.53 (±1.51)	3.21 (±1.02)	0.002
TG/HDL-C	3.31 (±2.5)	2.7 (±3.4)	0.014
ET-1 (pg/mL)	3.0 (±1.6)	2.2 (±1.0)	<0.001
NO (μmol/L)	35.7 (±15.2)	40.0 (±14.3)	<0.001
**Previous use of drugs**			
Aspirin	34 (11.5%)	28 (7.9%)	0.122
Beta-blockers	22 (7.4%)	27 (7.6%)	0.925
Statins	35 (11.8%)	34 (9.6%)	0.360
Diuretics	6 (2.3%)	8 (2.3%)	0.839
ACEI	36 (12.2%)	35 (9.9%)	0.354
ARB	33 (11.1%)	39 (11.0%)	0.958
CCB	45 (15.2%)	51 (14.4%)	0.776
Nitrates	13 (4.4%)	14 (4.0%)	0.781
Oral hypoglycemic	32 (10.8%)	34 (9.6%)	0.592
Insulin	5 (1.7%)	4 (1.1%)	0.787

*P-values were estimated from t-test for continuous variables and chi-square test for categorical variables*.

*LVSED, left ventricular end-systolic dimension; LVDED, left ventricular end-diastolic dimension; LVEF, left ventricular ejection fraction; HgB, hemoglobin; NLR, neutrophil to lymphocyte ratio; RDW, red cell distribution width; PDW, platelet distribution width; ALT, alanine transaminase; Crea, serum creatinine; eGFR, estimated glomerular filtration rate calculated by the abbreviated MDRD equation; UA, uric acid; CPR, C reative protein; HbA1c, glycosylated hemoglobin; TC, total cholesterol; TG, triglycerides; LDL-C, low-density lipoprotein cholesterol; HDL-C, high-density lipoprotein; Apo A1, apolipoprotein A1; Apo B, apolipoprotein B; Apo E, apolipoprotein E*.

In addition, current and cumulative use of medications (aspirin, beta-blockers, statins, diuretics, ACEI, ARB, CCB, nitrates, oral hypoglycemic, and insulin) were similarly distributed between groups (*P* > 0.05, Table 1).

### Prevalence of ED

ED was more common among patients with EOCAD than controls (57.8 vs. 31.1%, *P* < 0.001) (Table 2). Besides, the prevalence of ED of different degrees was higher in EOCAD subjects than controls (Table 2). The average IIEF-5 score was significantly lower in patients with EOCAD vs. controls (17.7 ± 6.0 vs. 21.6 ± 5.0, *P* < 0.001), suggesting more serious ED conditions in the EOCAD group comparing with controls (Table 2).

**Table 2:** Baseline erectile function evaluated by IIEF5 in EOCAD patients and controls.

	**EOCAD group**	**Control**	***P*-value**
IIEF5 Score	17.7 (±6.0)	21.6 (±5.0)	<0.001
No ED (22–25)	125 (42.2%)	244 (68.9%)	<0.001
Mild (17–21)	45 (15.2%)	42 (11.9%)	
Mild to moderate (12–16)	56 (18.9%)	48 (13.6%)	
Moderate (8–11)	54 (18.2%)	17 (4.8%)	
Severe (5–7)	16 (5.4%)	3 (0.8%)	

*P-values were estimated from t-test for continuous variables and Chi-square test for categorical variables*.

### Relationship Between Severity of Coronary Artery Atherosclerosis and ED

**Figure 1:**
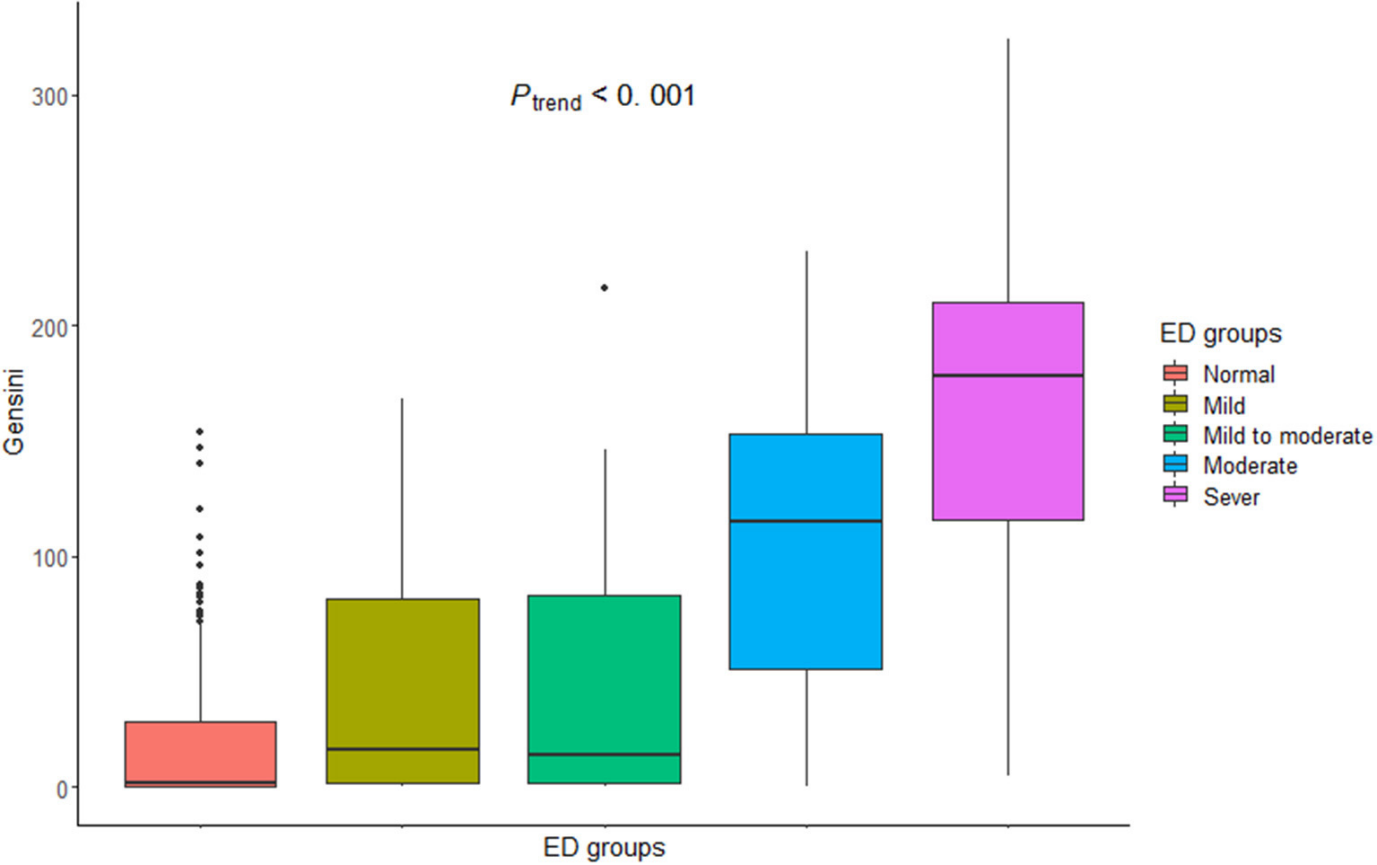
Gensini score among different groups of varying ED severity.

The mean of Gensini score, indicative of coronary artery atherosclerosis of varying severity, was higher in the EOCAD group vs. controls (82.4 ± 54.8 vs. 1.7 ± 2.6, *P* < 0.001). The Gensini score increased from the normal erectile function group to the severe ED group with means ± SDs of 48.87 ± 30.19 in the normal erectile function group, 81.18 ± 38.06 in the mild ED group, 79.80 ± 41.79 in the mild to moderate ED group, 135.20 ± 43.11 in the moderate ED group, and 179.00 ± 69.84 in the severe ED group (*P*_trend_ < 0.001, Figure 1).

### Logistic Regression Models for Prediction of ED

In univariate analysis, BMI, smoking, alcohol use, levels of UA, HbA1c, TG, HDL, ApoA, ApoE, non-HDL-C and TG/HDL, presence of EOCAD, and Gensini score were significantly associated with ED. In logistic regression analysis, adjusted OR for the presence of ED (EOCADs vs. controls) was 1.88 [95% confidence interval (CI), 1.12-3.18]. Other independent predictors for ED were BMI (OR: 1.17, 95%CI: 1.08-1.28), UA level (OR: 1.97, 95%CI: 1.64-2.39, per 58.94 umol/L increment), current smoking (OR: 10.48, 95%CI: 5.45-20.91), and ever smoking (OR: 2.11, 95%CI: 1.20-3.73) (Table 3). Interestingly, we further introduced vasoconstrictor index (ET-1) and vasodilator index (NO) into the multiple logistic regression model, and found that they attenuated the association between ED and EOCAD (OR: 1.54, 95% CI: 0.84-2.80), but did not materially affect the relationships of ED with other risk factors (Table 3). To understand this mediation effect, we next assessed the associations of ET-1 and NO with IIEF-5 and Gensini score, and expectedly found that these two vasomotor indexes were significantly correlated with IIEF-5 (r = −0.55 for ET-1, and r = 0.46 for NO, both *P* < 0.001) and Gensini score (r = −0.53 for ET-1, and r = 0.42 for NO, both *P* < 0.001) (Supplementary Figure 1).

**Table 3:** Associations between ED and risk factors by univariable analysis and multivariable logistic regression.

	**Univariable**		**Multivariable model 1**		**Multivariable model 2**	
**Variables**	**OR (95% CI)**	***P*-value**	**OR (95% CI)**	***P*-value**	**OR (95% CI)**	***P*-value**
Age (years)	0.98 (0.94-1.01)	0.154	–	–	–	–
BMI (kg/m^2^)	1.23 (1.16-1.30)	<0.001	1.17 (1.08-1.28)	<0.001	1.19 (1.09-1.32)	<0.001
**Smoking**						
No	1.00		1.00		1.00	
Ever	2.37 (1.58-3.57)	<0.001	2.11 (1.20-3.73)	0.010	2.19 (1.17-4.18)	0.016
Current	15.26 (9.63-24.75)	<0.001	10.48 (5.45-20.91)	<0.001	11.1 (5.06-25.55)	<0.001
**Drinking**						
No	1.00		1.00		1.00	
Mild	2.54 (1.77-3.67)	<0.001	0.96 (0.53-1.73)	0.892	0.83 (0.42-1.64)	0.602
Severe	1.72 (1.06-2.78)	0.028	0.89 (0.45-1.76)	0.743	0.64 (0.28-1.41)	0.277
History of hypertension	1.27 (0.92-1.76)	0.139	–	–	–	–
History of diabetes mellitus	1.54 (0.95-2.50)	0.078	0.66 (0.29-1.48)	0.319	0.78 (0.32-1.87)	0.589
History of dyslipidemia	0.92 (0.57-1.47)	0.734	–	–	–	–
History of atrial fibrillation	2.67 (0.94-8.65)	0.077	2.41 (0.45-13.05)	0.298	1.98 (0.34-11.75)	0.444
LVEF (%)	0.99 (0.98-1.01)	0.468	–	–	–	–
LVEDD (mm)	1.01 (0.99-1.04)	0.355	–	–	–	–
LVSED (mm)	1.01 (0.98-1.03)	0.614	–	–	–	–
HgB (g/L)	1.00 (0.99-1.01)	0.358	–	–	–	–
NLR	0.94 (0.85-1.02)	0.185	–	–	–	–
RDW (%)	1.06 (0.96-1.18)	0.273	–	–	–	–
PDW (%)	0.97 (0.92-1.02)	0.222	–	–	–	–
ALT (U/L)	1.00 (1.00-1.01)	0.313	–	–	–	–
Crea (μmol/L)	1.00 (0.99-1.00)	0.565	–	–	–	–
eGFR (ml/min/1.73m^2^)	1.00 (1.00-1.01)	0.345	–	–	–	–
UA (Per 58.94 umol/L increment)	2.18 (1.89-2.53)	0.000	1.97 (1.64-2.39)	<0.001	1.80 (1.46-2.26)	<0.001
CRP (mg/L)	1.01 (1.00-1.01)	0.080	1.00 (0.99-1.01)	0.427	1.00 (0.99-1.02)	0.694
HbA1c (%)	1.25 (1.09-1.44)	0.002	1.13 (0.88-1.44)	0.326	1.05 (0.79-1.39)	0.721
TC (mmol/L)	1.10 (0.96-1.25)	0.166	–	–	–	–
TG (mmol/L)	1.14 (1.04-1.26)	0.005	–	–	–	–
HDL (mmol/L)	0.38 (0.21-0.70)	0.002	–	–	–	–
LDL (mmol/L)	1.05 (0.91-1.21)	0.474	–	–	–	–
ApoA (g/L)	0.33 (0.15-0.68)	0.003	0.63 (0.21-1.92)	0.421	0.63 (0.18-2.22)	0.472
ApoB (g/L)	1.49 (0.84-2.64)	0.174	–	–	–	–
ApoE (g/L)	1.01 (1.01-1.02)	0.003	1.01 (0.99-1.03)	0.570	1.02 (1.00-1.05)	0.144
NHDL-C (mmol/L)	1.14 (1.01-1.30)	0.041	0.93 (0.73-1.17)	0.536	0.87 (0.65-1.15)	0.323
TG/HDL	1.09 (1.03-1.17)	0.005	0.97 (0.85-1.09)	0.585	0.94 (0.82-1.09)	0.422
ET-1 (pg/ml)	2.50 (2.11-3.00)	<0.001	–	–	1.97 (1.49-2.64)	<0.001
NO (μmol/L)	0.92 (0.91-0.94)	<0.001	–	–	0.93 (0.90-0.95)	<0.001
EOCAD	3.03 (2.20-4.20)	<0.001	1.88 (1.12-3.18)	0.017	1.54 (0.84-2.80)	0.159
Gensini score	1.02 (1.02-1.03)	<0.001	–	–	–	–

*Factors with P < 0.1 in univariable analysis were included for multivariable logistic regression. TG/HDL-C was included in multivariable logistic regression instead of TG or HDL-C separately because of obvious interaction. Gensini score was excluded in multivariable logistic regression because of obvious colinearity with EOCAD*.

### Improvement of ED After Coronary Revascularization and the Impact of Beta-Blockers

**Figure 2:**
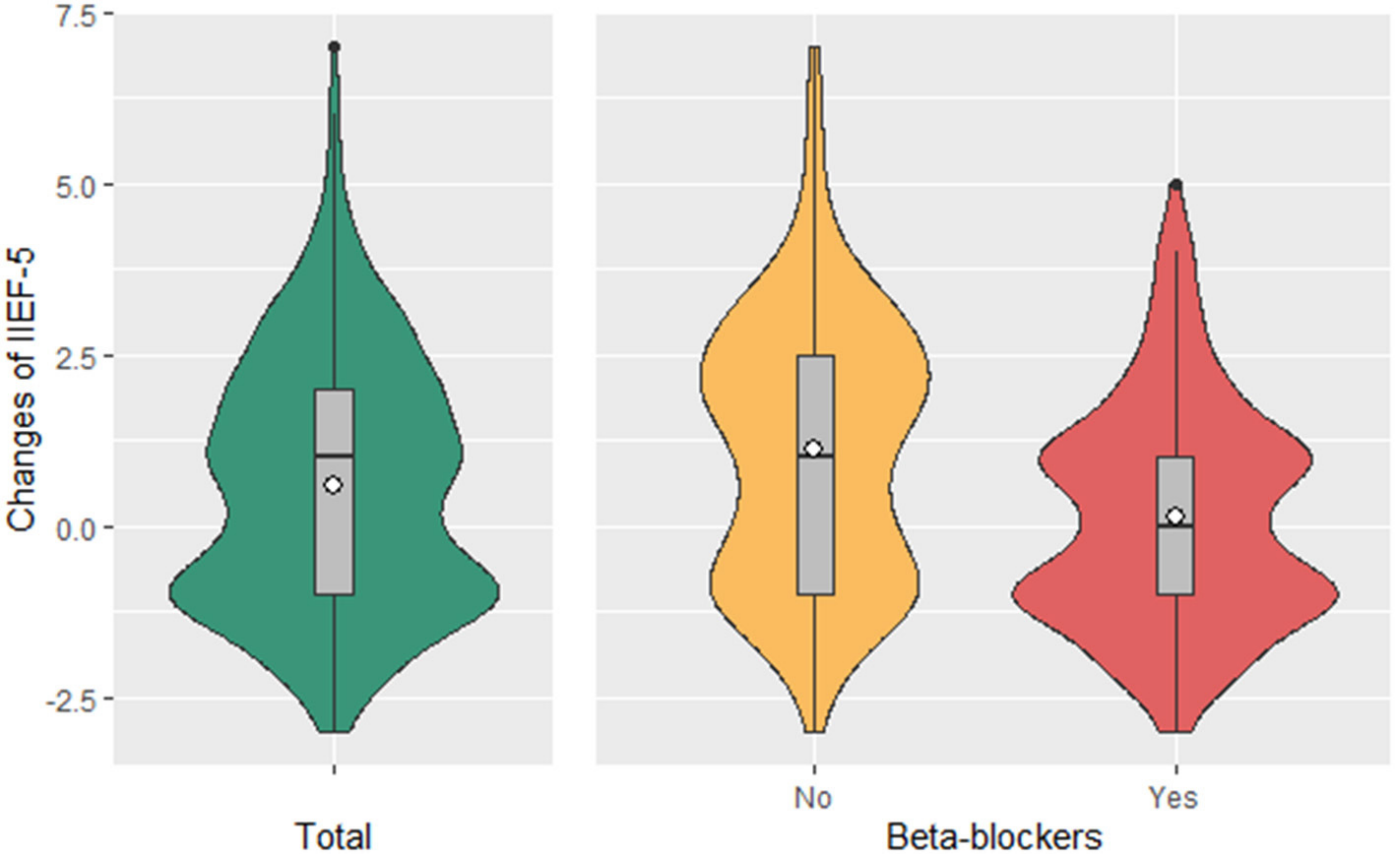
Changes in IIEF-5 Score in patients with or without beta blocker use. Mean change in IIEF-5 in patients on beta blockers (157, 53.0%) was 0.12, *P* = 0.09; mean change in IIEF-5 in patients not on beta blockers (139, 47.0%) was 1.11, *P* < 0.001. *P*-values were estimated from the linear mixed-effects models after adjustment for BMI, smoking, alcohol use, UA, CRP, HbA1c, Apo-A, Apo-E, the TG and HDL-C ratio, and the history of diabetes mellitus and atrial fibrillation. ANOVA *P* < 0.001 for the comparison between patients with or without beta blocker use, adjusted for BMI, smoking, alcohol use, UA, CRP, HbA1c, Apo-A, Apo-E, the TG and HDL-C ratio, and the history of diabetes mellitus and atrial fibrillation.

Among EOCAD patients, IIEF-5 score significantly elevated 1 year after coronary revascularization relative to baseline (18.24 ± 5.00 vs. 17.65 ± 6.00, *P* < 0.001; Figure 2). Patients were further divided into subgroups according to the use of beta-blockers, with 157 patients (53.0%) receiving beta-blockers after coronary revascularization. Use of other medications (aspirin, clopidogrel, statins, diuretics, ACEI, ARB, CCB, nitrates, oral hypoglycemic and insulin) was similarly distributed between groups on or not on beta-blockers after discharge (Supplementary Table 1). In this subgroup analysis, the IIEF-5 scores after revascularization significantly increased in patients not on beta-blockers (18.71 ± 4.84 vs. 17.59 ± 6.05, *P* < 0.001), while stayed unchanged in patients on beta-blockers (17.82 ± 5.12 vs. 17.70 ± 5.98, *P* = 0.09) as compared with those at baseline (Figure 2).

## Discussion

In the present study, we found ED was more prevalent in EOCAD patients than that in controls. While two vasomotor indexes (i.e., ET-1 and NO) attenuated the association between ED and EOCAD. Among EOCAD patients, we found the IIEF-5 score elevated 1 year after coronary revascularization. Of note, this beneficial effect of revascularization did not appear in patients using beta-blockers.

ED is considered to affect a large number of men (10). However, the accurate incidence and prevalence of ED are uncertain because of considerable limitations when conducting epidemiological studies of sexual function. The diagnosis of ED is mainly based on a questionnaire and therefore subjective, and because it is not life-threatening, individuals who suffer it tend not to seek treatment. Studies have estimated that ED incidence ranges from 31.7 to 80% in different age groups (11, 12), and ED prevalence ranges from 12.9 to 52% in different populations (13–16). The Massachusetts male aging study (MMAS) was the first population study to show that the prevalence of ED was 52% in men aged 40–70 years (13). In young males, ED is a particularly important issue because they are sexually active. The National Health of Social Life Survey (NHSLS) showed an ED prevalence of 50% in men aged 18-59 years (14). The incidence of ED in young males is underestimated, and ED has become increasingly common because of the increased stress, anxiety, depression, and sympathetic tone in modern society (6). In this study, the prevalence of ED in young controls aged ≤ 45 years without CAD was 31.1%, which was similar to that reported previously in the general population for that age group.

During the last two decades, somatic factors, especially atherosclerotic lesions, have been recognized to play a major role in the development of ED. The landmark MMAS (13) and other recent studies (17–19) have raised awareness of the significant association between cardiovascular disease and the occurrence of ED and its severity in a general population of men. Secondary to systemic atherosclerosis and concomitant endothelial dysfunction, which can restrict blood flow to the corpus cavernosum during erection, the prevalence of sexual dysfunction is higher in patients with cardiovascular disease as compared with the general population. Likewise, the prevalence of sexual dysfunction in this study is higher in EOCAD patients compared with controls. Furthermore, the severity of ED is significantly related to the extent of coronary lesion severity and atherosclerotic burden revealed by the Gensini score.

Both cardiovascular disease and ED share common causal factors, such as older age, cigarette smoking, lack of exercise, hypertension, obesity, diabetes mellitus, dyslipidemia, hyperuricemia, and metabolic syndrome (20), all of which might lead to systemic atherosclerosis and concomitant impairment of the endothelial function (21). In response to humoral, neural, and mechanical stimuli, the vascular endothelium releases a variety of factors including NO and ET-1 to regulate the contractile and relaxatory homeostasis. Imbalance in the production of vasodilator and vasoconstrictor agents may contribute to the onset of endothelial dysfunction (22). Endothelial-derived NO plays a crucial role in the physiology of erection, including initiating and maintaining intracavernous pressure increase, penile vasodilatation, and penile erection that are dependent on activation of guanylate cyclase and the subsequent production of cGMP by NO in smooth muscle cells (23, 24). Therefore, incidence and severity of the endothelial dysfunction, which is also one of the earliest landmarks in the development of atherosclerotic lesions and a predictor of cardiovascular outcome, maybe a pathophysiologic mechanism underlying both ED and CVD, forming a unifying link between these two conditions (25). In clinical practice, ED might even be considered as an early clinical manifestation of endothelial dysfunction and a harbinger of undesirable cardiovascular events, especially in the young male (26). In our current study, the association of EOCAD with ED is dependent on endothelial dysfunction. EOCAD was an independent predictor of ED only in the multivariate model without the adjustment of endothelial dysfunction, while not when parameters of ED were included. It indicated that endothelial dysfunction may be an underlying common cause of EOCAD and ED.

Of note, a few studies have suggested that low levels of testosterone were associated with the development and severity of EOCAD in males (27). Such an association could not be explored in the present cohort because of the lack of data on testosterone levels and warrants further investigation.

Another possible explanation for ED in EOCAD patients may be the fear or anxiety of developing coital angina during sexual activity, particularly at orgasm, during which the maximal energy expenditure occurs, and oxygen uptake reaches its peak. Hence, sexual activity can trigger coital angina *via* dynamic and transient increased myocardial oxygen demand secondary to an increase in heart rate, blood pressure, and the effects of arousal (28). Because revascularization helps to improve myocardial blood flow and the tolerance for maximal energy expenditure, it may eliminate coital angina and thereby improve erectile function. In this study, IIEF-5 score was significantly increased after coronary revascularization, indicating the amelioration of ED symptoms.

Previous studies indicated that beta-blockers may cause ED (29). The potential mechanisms underlying the impact of beta-blockers on sexual function may include inhibition of the sympathetic nervous system, which is involved in the integration of erection, emission and ejaculation, impairment of vasodilation of the corpora cavernosa, effects on luteinizing hormone and testosterone secretion and a tendency to produce sedation or depression (30). However, beta-blockers are the first-line agent for angina pectoris and also preferably used in young patients with hypertension due to the predominant increase in sympathetic nerve activity in this population. In the present study, beta-blockers adversely affected ED improvement conferred by coronary revascularization. Their apparent disadvantageous effects on erectile function and quality of life for the young, calls for into question considering beta-blockers as the first-line option after coronary revascularization in the EOCAD patients with ED.

## Limitations

Interpretation and generalizability of the findings presented are limited by the observational, single-center design of this study in a Chinese population of men. Larger, multicenter studies with greater ethnic diversity are warranted to validate the findings presented. In addition, we did not measure the testosterone level, which may be a confounder for the association between ED and EOCAD.

## Conclusions

In our study, we revealed that ED cannot be neglected in young men with active sexual desire, with an especially higher prevalence in EOCAD patients. Endothelial dysfunction may be a pathophysiologic mechanism underlying both ED and EOCAD. Coronary revascularization appeared beneficial to the improvement of ED, while this improvement did not appear in patients using beta-blockers. Beta-blockers for antianginal therapy after revascularization should be used with extra caution after fully informing the patients who have EOCAD and ED.

## Data Availability Statement

The original contributions presented in the study are included in the article/Supplementary Material, further inquiries can be directed to the corresponding author/s.

## Ethics Statement

The studies involving human participants were reviewed and approved by the ethical committee of the Zhongshan Hospital. The patients/participants provided their written informed consent to participate in this study.

## Author Contributions

YD, KY, and JG conceived and designed the study. YD, ZM, and SZ conducted the statistical analysis and drafted the manuscript. KY and JG had full access to all of the data and took responsibility for the integrity of the data and the accuracy of the data analysis. All authors participated in the interpretation of the results and revision of the manuscript and read and approved the final manuscript.

## Conflict of Interest

The authors declare that the research was conducted in the absence of any commercial or financial relationships that could be construed as a potential conflict of interest.
