# Supplementary material for: Sexual Dysfunction and the Impact of Beta-Blockers in Young Males With Coronary Artery Disease
Source: Front Cardiovasc Med. 2021 Jul 21;8:708200. doi: 10.3389/fcvm.2021.708200 (PMC8333273; doi:10.3389/fcvm.2021.708200)

**Table S1.** Medication use after discharge in EOCAD groups according to use or not of beta-blockers

|  | **On beta-blockers after discharge (N=139)** | **Not on beta-blockers after discharge (N=157)** | ***P* value** |
| --- | --- | --- | --- |
| Aspirin | 34 (11.5%) | 28 (7.9%) | 0.122 |
| Clopidogrel | 22 (7.4%) | 27 (7.6%) | 0.925 |
| Statins | 35 (11.8%) | 34 (9.6%) | 0.360 |
| Diuretics | 6 (2.3%) | 8 (2.3%) | 0.839 |
| ACEI | 36 (12.2%) | 35 (9.9%) | 0.354 |
| ARB | 33 (11.1%) | 39 (11.0%) | 0.958 |
| CCB | 45 (15.2%) | 51 (14.4%) | 0.776 |
| Nitrates | 13 (4.4%) | 14 (4.0%) | 0.781 |
| Oral hypoglycemic | 32 (10.8%) | 34 (9.6%) | 0.592 |
| Insulin | 5 (1.7%) | 4 (1.1%) | 0.787 |

*P* values were estimated from chi-square test.

**Figure S1.** Correlations of ET-1 and NO with IIEF-5 and Gensini score.


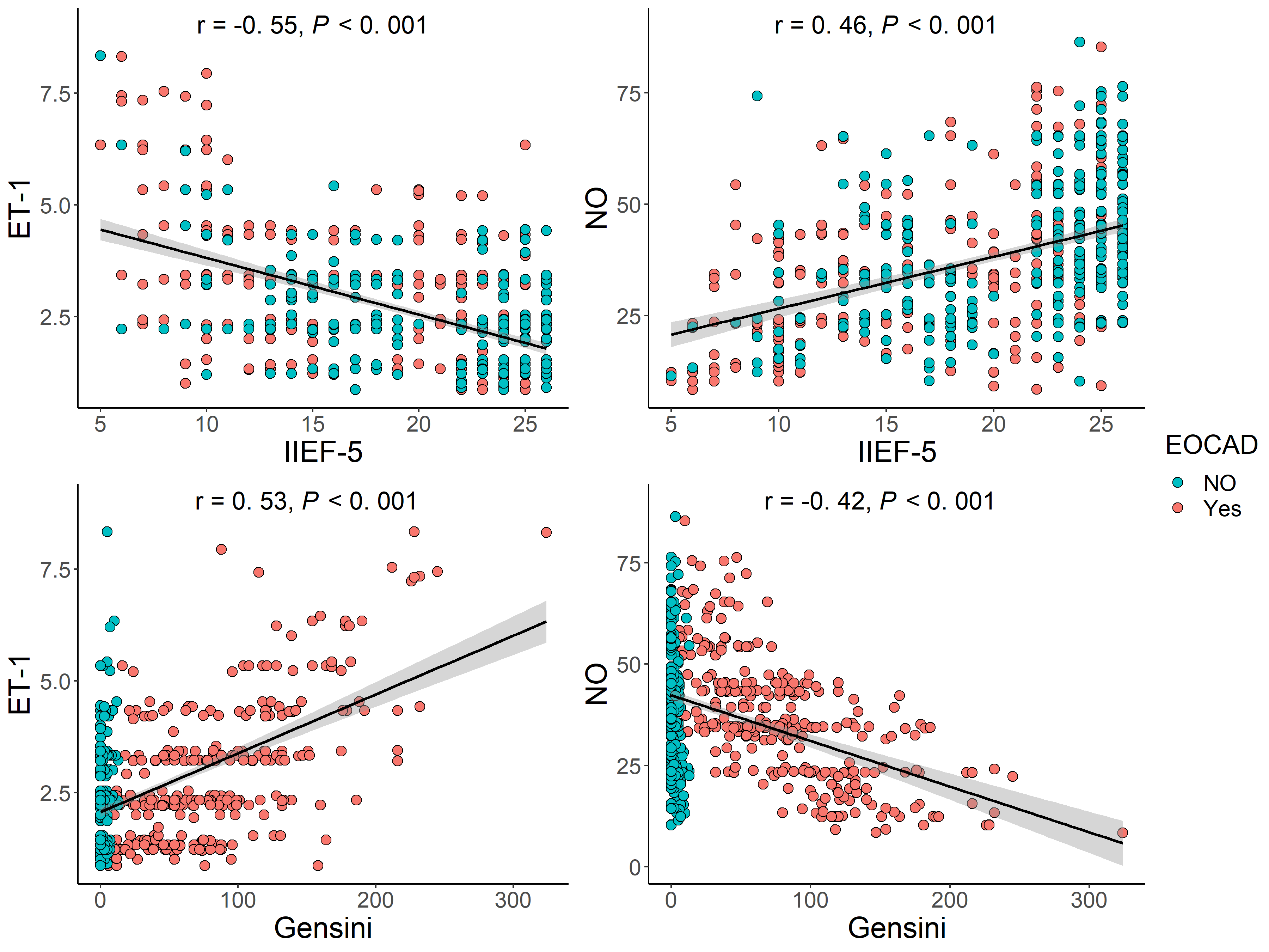

Supplement: Supplementary file 1 [file Data_Sheet_1.docx]
